# Impact after 10-year use of pneumococcal conjugate vaccine in the Brazilian national immunization program: an updated systematic literature review from 2015 to 2020

**DOI:** 10.1080/21645515.2021.1879578

**Published:** 2021-03-18

**Authors:** Adriana Guzman-Holst, Eliana de Barros, Pilar Rubio, Rodrigo DeAntonio, Otavio Cintra, Ariane Abreu

**Affiliations:** a GSK, Panama City, Panama; b GSK, Rio de Janeiro, Brasil; c Centro de Vacunación Internacional S.A. CEVAXIN, Panama City, Panama; d GSK, Wavre, Belgium; e Instituto Nacional de Cardiologia, Rio de Janeiro, Brasil

**Keywords:** Streptococcus pneumoniae, vaccination, PCV, Brazil, invasive pneumococcal disease, Pneumonia, acute otitis media, nasopharyngeal carriage, impact

## Abstract

In 2010, a 10-valent pneumococcal non-typeable *Haemophilus influenzae* protein D conjugate vaccine (PHiD-CV) was introduced in the Brazilian national immunization program; the 3 + 1 dose schedule was replaced by a 2 + 1 dose schedule in 2016. This systematic review presents the latest published evidence (2015–2020) on the impact after 10-year use of PHiD-CV in Brazil from a total of 29 publications. Overall, the PHiD-CV program had a positive impact on the morbidity and mortality associated with invasive pneumococcal disease (IPD), pneumonia and acute otitis media (AOM) in children <5 years-old. A reduction in the vaccine-type invasive disease was observed in all-ages; suggesting indirect protection unvaccinated older children and adults. The occurrence of non-vaccine type disease was evidenced in some studies. Higher vaccination coverage is required at national and state level for sustained population impact. Given the change in the vaccination schedule and the dynamics of pneumococcal disease epidemiology, continuous surveillance is warranted.

GSK Study identifier: HO-18-19438

## Introduction

*Streptococcus pneumoniae* causes invasive pneumococcal disease (IPD) such as meningitis, bacteremia, pneumonia and sepsis, and noninvasive diseases such as non-bacteremic pneumonia and acute otitis media (AOM), both of which result in high morbidity and mortality worldwide.^1^ Pneumococcal conjugate vaccines (PCVs) have been introduced in the national immunization program for children in several countries.^1^ In 2010, the 10-valent pneumococcal non-typeable *Haemophilus influenzae (*NT*Hi)* protein D conjugate vaccine (PHiD-CV, Synflorix™, GSK Vaccines) was introduced in the Brazilian routine childhood immunization program with three primary doses given to children at 2, 4 and 6 months of age followed by a booster dose at 12–15 months of age (3 + 1 schedule).^2^ In addition to the routine program, Brazil provided a catch-up program for infants with incomplete schemes to get two primary doses for infants 7–11 months of age with a booster dose later at 12–15 months of age, and one dose for children 12–23 months of age.^2,3^

In 2016, 6 years after introducing PHiD-CV in the routine immunization program, Brazil revised its recommendation for PHiD-CV; the 3 + 1 vaccination schedule (at 2, 4 and 6 months of age followed by a booster dose at 12–15 months of age) was replaced by a 2 + 1 vaccination schedule (at 2 and 4 months of age followed by a booster dose at 12 months of age and a catch-up of one dose for non-vaccinated children from 12 months to 4 years of age).^4^

In countries with economies in transition like Brazil, most cases of pneumococcal disease and deaths among children below 5 years occur in the first year of life, with a peak in disease incidence before 6 months of age.^5^ The timing of onset of disease and death is critical in determining the vaccine schedule.^6^ The World Health Organization (WHO) recently recommended the PCV schedule of three primary doses without a booster or, as an alternative, two primary doses with a booster.^1^ Evidence from immunogenicity studies, as well as clinical trials and observational studies of nasopharyngeal carriage and IPD, have been systematically reviewed and results indicate that both schedules (3 + 1 and 2 + 1) work well. Either schedule can be implemented based on local epidemiology or programmatic considerations, but it is key to achieve a high three-dose (2 + 1) coverage to reach an adequate overall level of protection and herd effect.^7^

In Brazil, the national coverage of the PHiD-CV primary series (from 2010 to 2015 primary series comprised three doses, but from 2016 onwards the primary series comprised two doses) was >80% from 2011 to 2019 and >70% for the booster dose from 2013 to 2019.^8^ However, in 2019 vaccine uptake declined in relation to previous years and disparities in PHiD-CV coverage rates in the different regions, cities, and municipalities of Brazil were reported.^3^ This is important to address as vaccine uptake in children impacts the size of herd protection effects.^9^

A recent literature review, with evidence until 2015 (5 years after PHiD-CV introduction), reported an overall positive impact on pneumococcal disease burden 5 years after PHiD-CV introduction in Brazil.^3^ Additionally, indirect protection against vaccine-type IPD and pneumonia hospitalizations in unvaccinated subjects was observed in individuals of older age groups.^3^

The current systematic literature review aims to provide an update on the direct and indirect impact of the use of PHiD-CV immunization on the burden of IPD, pneumonia, AOM, and pneumococcal nasopharyngeal carriage, in the Brazilian population, since the schedule change and the last review^3^ which provided evidence of a positive early impact on the burden of pneumococcal disease in Brazil.

## Methods

We conducted an update of a previously published systematic review,^3^ according to guidelines in the Cochrane Handbook for Systematic Reviews of Interventions^10^ and Preferred Reporting Items for Systematic Literature Reviews and Meta-Analyses (PRISMA),^11^ to obtain relevant information using a reproducible, robust and transparent methodology.

### Search sources and strategy

The following online databases were searched: Medline (via PubMed), Web of Science, Scopus, Scientific Electronic Library Online (SciELO), and Latin American & Caribbean Health Sciences Literature (Lilacs). We also searched other sources such as congress proceedings published from June 2015 through April 2018, including the International Symposium on Pneumococci and Pneumococcal Diseases (ISPPD) (2016, 2018), the Congreso Latinoamericano de Infectología Pediátrica (SLIPE), Interscience Conference of Antimicrobial Agents and Chemotherapy (ICAAC), Annual Meeting of the European Society for Pediatric Infectious Diseases (ESPID), World Congress of the World Society for Pediatric Infectious Diseases (WSPID), International Congress on Infectious Diseases (ICID), IDWeek, Congresso Brasileiro de Pediatria, Congresso Brasileiro de Pneumologia Pediátrica, and Congresso Brasileiro de Infectologia Pediátrica.

In addition, we searched epidemiological surveillance databases, when available, from the local surveillance systems such as Sistema de Informação de Agravos de Notificação (SINAN) (via the Brazilian government open-access public health database system [DATASUS]), Centro de Vigilância Epidemiológica da Secretaria de Estado da Saúde de São Paulo (CVE, São Paulo State), and Sistema de Informação do Programa Nacional de Imunizações (SI-PNI).

The search strategy was developed using both free-text and MeSH terms. Search terms for the different databases were combined using Boolean operators (e.g. vaccine OR otitis OR pneumonia OR invasive disease OR carriage OR herd protection AND Brazil). Details of the search strategy are provided in Supplementary material (Supplementary Tables 1–6). All searches in this update were restricted by publication date from May 2015 through May 2020, to capture relevant evidence after the date of search in Moreira et al.^3^ Articles published in English, Spanish and Portuguese were included in this review and the geographic scope was restricted to Brazil.

### Article selection and data extraction

Identified publications were screened in two phases by two reviewers using the inclusion and exclusion criteria provided in Table 1. The first phase included a screening of titles and abstracts of all publications followed by a second phase which included reviewing the full-text of articles. All discrepancies were discussed between both reviewers and in case of disagreement about article inclusion a third reviewer was consulted.

**Table 1.** Inclusion and exclusion criteria

	Inclusion criteria	Exclusion criteria
Population	Studies done in human subjects of all ages	Non-human studies All others
Intervention	PHiD-CV vaccine	All others were excluded
Comparator	All	None were excluded based on comparator
Outcome	Epidemiological parameters Prevalence/incidence Effectiveness of vaccination on invasive disease, pneumonia, otitis media, nasopharyngeal carriage, serotype distribution, or herd protection in all age groups	Vaccine immunogenicity or safety studies Studies evaluating treatments such as antibiotics; studies of virus etiology only; studies conducted among populations with chronic diseases not representative of the general population.
Study design	Primary research* Randomized controlled trials (RCTs) Non-randomized studies Observation studies (cohort studies, case-control studies, pre-/post-vaccine introduction time series) Surveillance reports (unpublished and published) Publications/reports from global, regional and local published and unpublished data (if possible)	Non-primary research Reviews** Meta-analysis Public health programs or recommendations Transmission modeling studies Cost-effectiveness or health economics studies Case reports Meta-analysis Letter to editor Newspaper Editorial Comment Opinion paper Studies whose analysis period was unspecified.
**Limits**		
Publication date	May 2015 – May 2020	Articles published outside of dates considered eligible for inclusion
Geographic scope	Brazil	All other countries/regions
Language	English, Spanish and Portuguese	Articles published in any other language besides English, Spanish and Portuguese

* Reference lists of all articles were manually screened for additional relevant original articles (as deemed necessary by the reviewer)

**Review articles from the search were excluded but bibliographies of reviews were manually screened for additional relevant original articles.

Relevant information about each article, established *a priori*, was extracted, including journal and year of publication, study setting, study objectives, study design, study period (e.g., pre- and post-vaccination periods), sample size, study population, clinical outcome, measure of impact of vaccination (effectiveness or percentage incidence, number of cases changes).

### Risk of bias and quality assessment

First, the risk of bias for observational studies was independently assessed by two reviewers through the Strengthening the Reporting of Observational Studies in Epidemiology (STROBE) checklist,^12^ modified according to Sanderson et al.^13^ and Fowkes and Fulton.^14^ An algorithm was used to estimate the overall risk of bias considering five criteria: methods for selecting study participants, methods for measuring exposure and outcome variables, methods to control confounding, design-specific sources of bias, and statistical methods (Supplementary Table 7).

Second, the quality of overall evidence and strength of recommendation was evaluated using the Grading of Recommendations, Assessment, Development, and Evaluations (GRADE) criteria for all eligible publications.^15^ The quality of evidence was rated for each outcome, taking into account the risk of bias from the STROBE assessment, consistency, directness, precision, publication bias, the magnitude of the effect, dose-response, and confounders (Supplementary Figure 1). We applied a score system based on GRADE criteria which shows how we rated the quality of each individual study (*high* to *very-low*). Studies that have observational designs automatically fall under the *low*-quality category, but we further categorized the studies into *very-low* to *low* (Supplementary Table 8). The resultant strength of recommendation (*strong/medium/weak*) was informed by the quality of the evidence (explained previously), the balance of outcomes (desirable versus undesirable) and value magnitude. The quality assessment of abstracts was not performed as study details were not adequately reported.

### Analyses and reporting

A descriptive analysis was conducted to summarize each of the disease endpoints established in the inclusion criteria. Statistical heterogeneity between studies was assessed using I-square (I^2^) statistic, with values less than 40% indicating an insignificant level of heterogeneity, values between 40% and 60% indicating a moderate level of heterogeneity and values over 60% indicating substantial heterogeneity.^16^ In case of significant heterogeneity (>60%) of results or inconsistent methods used (i.e. study designs) across studies, the outcome estimates were not pooled in a meta-analysis and a description of individual studies was provided.

## Results

### Description of included studies

**Figure 1.**
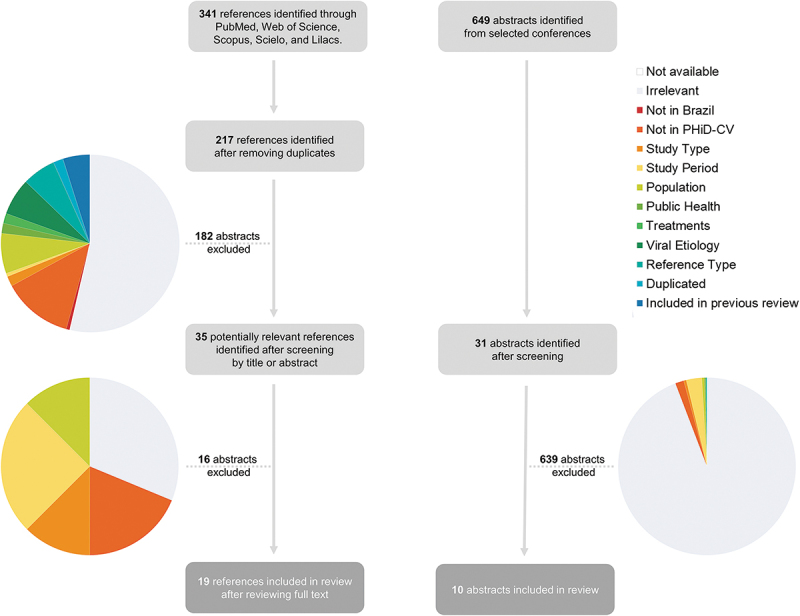
PRISMA flow chart of articles and conference abstracts identified for inclusion in the review.

**Figure 2.**
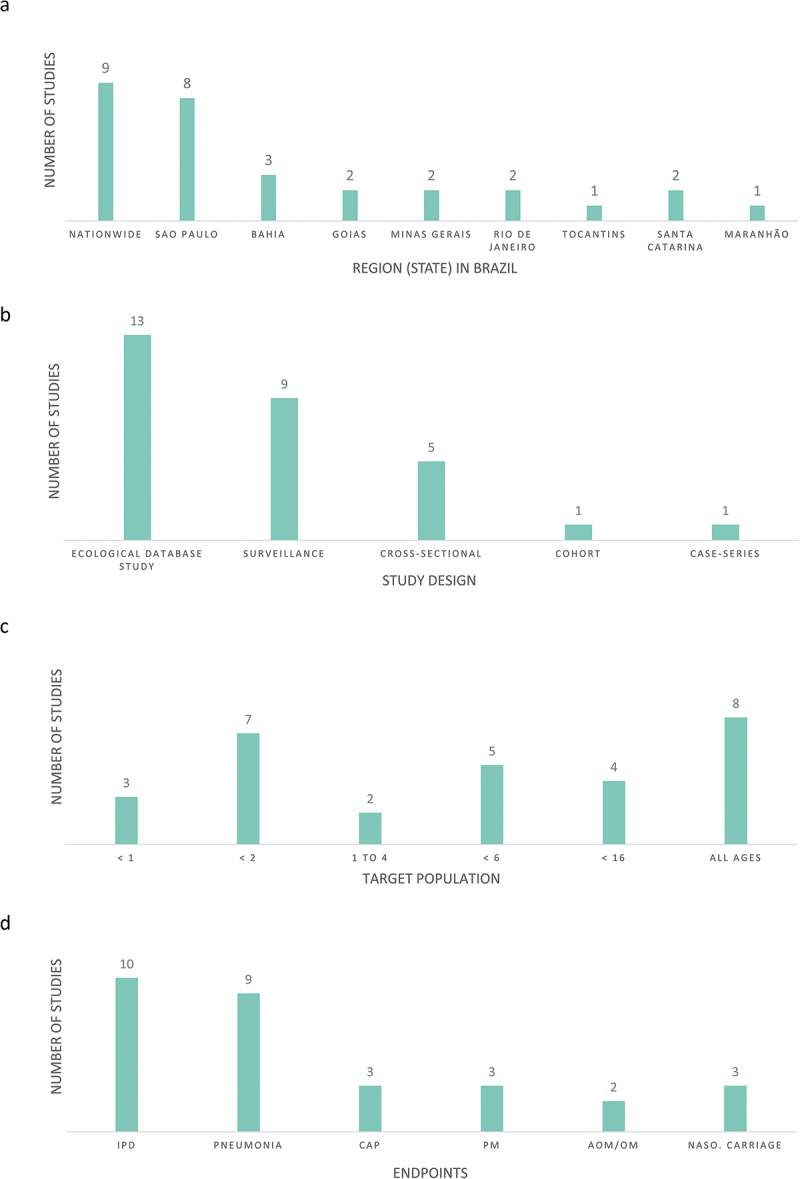
Distribution of studies by (A) Region (state) in Brazil (N = 29)*, (B) Study design (N = 29), (C) Target population (N = 29), and (D) Outcome (N = 29)*

AOM, acute otitis media; CAP, community-acquired pneumonia; IPD, invasive pneumococcal disease; Naso., nasopharyngeal; OM, otitis media. *Total N = 30 because in 1 publication >1 outcome was reported.

A total of 19 papers (out of 341 full-text journal publications identified from the online databases)^17–35^ and 10 abstracts (out of 649 from relevant conference proceedings)^36–45^ were included in the review (Figure 1). A summary of study characteristics is presented in Figure 2 and individual study details are provided in Table 2.

**Table 2.** Impact of PHiD-CV on IPD, pneumonia, CAP, AOM and nasopharyngeal carriage in Brazil

								Incidence or Percent Cases (% or #s)	
Reference	Geographic Area	Study Design	Study Period (years after vaccine introduction)	Sample Size	Age	Outcome	Effectiveness/Impact or Percent Change (%)	Pre-Introduction	Post-Introduction
**IPD**									
Andrade et al. 2015 (abstract)^36^	Nationwide	Ecological Database Study	2008-2013 (3 years)	Not Stated	< 2 years	PMIPD	-44.4% (95%CI: -72.5; -15.8; p<0.002)		
Jarovsky et al. 2016 (abstract)^40^	Sao Paulo (Sao Paulo State)/ Uberlandia (Minas Gerais State)	Surveillance	Pre: Jan 2000-2009Post: 2010 to Dec 2013 (3 years)	445 IPD episodes	< 16 years	IPD		Cases: • 60% (<2 years) • 23% (2-5 years) • 16% (>5 years)	Cases: 42% (<2 years) 28% (2-5 years) 25% (>5 years)
Yoshioka et al. 2016 (abstract)^45^	Sao Paulo State	Surveillance	Pre: 2005-2010Post: 2011-2016 (6 years)	205 patients	< 15 years	IPD		Cases: 150 Diagnosis: • Pneumonia (66.7%) • Bacteremia (19.3%) • Meningitis (10.7%)	Cases: 55 Diagnosis: • Pneumonia (67.3%) • Bacteremia (14.5%) • Meningitis (12.7%)
Leite et al. 2016^26^	Salvador (Bahia State)	Case-series	Post: Jul 2010 to Dec 2013 (3 years)	82 eligible cases	All ages (stratified)	IPD	• 11/14 (78.6%) *Sp* isolated strains belonged to VT		• Pneumococcal meningitis (n = 64, 78.1%) • Bacteremic pneumococcal pneumonia (n = 12, 14.6%) • Bacteremia (n = 6, 7.3%)
Medeiros et al. 2016^27^	Sao Paulo State (University Hospital)	Cross-sectional	1998 to 2013 (3 years)	332 isolated pneumococcal strains in patients with IPD	All ages (stratified)	IPD		n = 205 (61.7%)	n = 46 (13.9%)
Medeiros et al. 2017^28^	Sao Paulo State	Surveillance	1998 to 2013 (3 years)	796 patients (isolates)	All ages (stratified)	IPD		n = 479	n = 95 cases
Brandileone et al. 2018^20^	Nationwide	Surveillance	Three periods (3 years and 5 years): • Pre- PHiD-CV (Jan 2005-Dec 2009) • Early post- PHiD-CV (Jan 2010-Dec 2013) • Late post- PHiD-CV (Jan 2014-Dec 2015)	8,971 IPD isolates	All ages (stratified)	VT IPDNVT IPD			Reduction in VT-IPD meningitis cases: • 83.4% (2-4 years) • 47.4% (>65 years)
Jarovsky et al. 2017 (abstract)^42^	Sao Paulo State	Surveillance	Pre: Jan 2000-2009Post: 2010- Apr 2017 (7 years)	561 patients (440 in analysis)	All ages (stratified)	IPD	IPD decreased from 35.9 to 30.3 cases/year (-15.6%) at all ages		
Cassiolato et al. 2018^23^	Nationwide	Surveillance	Pre: 2005 - 2009 Post: 2011 - 2017	673 19A isolates (399 19A MLST)	All ages (stratified)	IPD		19A: 2005-2009: 2.8%	19A: 2011-2015: 7.0%2016-2017: 16.4%
Berezin et al. 2020^19^	Nationwide	Surveillance	Pre:2005-2009 Post:2011-2015	260 IPD patients	< 17 years	IPD hospitalizations and mortality	260 patients with IPD and positive pneumococcal isolates were identified (198 during the pre-PCV10 period). When comparing both periods, hospitalizations were reduced from 20 cases to 5 cases per 10,000 pediatric admissions (p < 0.0001). Likewise, fatalities reduced from 6.6 to 2.0 cases per 10,000 pediatric admissions (p < 0.0001).		
**Pneumonia**									
Kupek et al. 2016^24^	Santa Catarina State	Ecological Database Study	Pre: 2006-2009Post: 2010-2013 (3 years)		< 1 year	Pneumonia mortality	Reduction mortality of 11%	29.69/100,000	23.40/100,000
De Oliveira et al. 2017 (abstract)^38^	Nationwide (focus on North region and Ampa State)	Ecological Database Study	Pre: 2006Post: 2016 (6 years)	N/A	< 1 year	Pneumonia mortality		Child Mortality Rates by region: • Norte (1.1) • Nordeste (1.08) • Centro-Oeste (0.99) • Sudeste (0.69) • Sul (0.60) North Region: • Amapaá (1.91) • Acre (1.81) • Tocantins (1.33) • Amazonas (1.05) • Paraá (1.01) • Roraima (0.80) • Rondoônia (0.72)	Child Mortality Rates by region: • Norte (1.66) • Nordeste (1.24) • Centro-Oeste (0.98) • Sudeste (0.68) • Sul (0.47) North Region: • Amapaá (1.43) • Acre (3.97) • Roraima (3.19) • Amazonas (1.93) • Rondoônia (1.78) • Paraá (1.34) • Tocantins (0.47)
Kurum et al. 2017^25^	Nationwide	Ecological Database Study	2003 to 2013 (3 years)	Unclear	< 2 years	Pneumonia hospitalizations	Reduction: • 0-<12 months: 10% (95%CI: 4–19) • 12–23 months): 7% (95%CI: 1–10) • 24–59 months: 11% (95%CI: 4–13)		
Costa et al. 2015 (abstract)^37^	Maranhaão State	Ecological Database Study	Pre: 2008 - 2010Post: 2011- 2013 (3 years)	47,429 pneumonia hospitalization (1-4 years)	1–4 years	Pneumonia hospitalizations	Reduction of 10.1% in hospitalizations (not significant)	Hospitalizations: 24,981 (52.7%)Deaths:43 (48.9%)	Hospitalizations: 22,448 (47.3%)Deaths: 45 (51.1%)
Marani et al. 2015 (abstract)^43^	Tocantins State	Ecological Database Study	Pre: 2008 - 2010Post: 2011- 2013 (3 years)	14,938 pneumonia hospitalization (1-4 years)	1–4 years	Pneumonia hospitalizations	Reduction of 28% in deaths by pneumonia (significant)	Hospitalizations:Pre: 8,128 (54.4%)Deaths: 107 (58.2%)	Hospitalizations:Post: 6,810 (45.6%)Deaths: 77 (41.8%)
Andrade et al. 2017^17^	Nationwide	Ecological Database Study	Pre: Jan 2005 -Dec 2009Post: Jan 2011 - Dec 2015 (5 years)	78,727,692 records analyzed (7,829,895 (9.9%) for pneumonia)	< 6 years	Pneumonia hospitalizations (herd immunity)	Impact: <12 months: -26.5 (95%CI: -35.5; -17.5); p=0.001 2-4 years: -21.5 (95%CI: -29.8; -13.2); p=0.002		
Oliveira et al. 2017 (abstract)^44^	Nationwide	Ecological Database Study	Pre: 2006-2010Post: 2011-2015 (4 years)	N/A	< 6 years	Pneumonia mortality	Reduction of 21,05% deaths by pneumonia	Child Mortality Rates:Pre: 0,746 deaths/1000 live births	Post: 0,589 deaths/1000 live births
Vieira et al. 2018^35^	Santa Catarina State	Ecological database study	Pre: 2006 - 2009 Post: 2010 - 2014	75,891 children admitted to hospital with pneumonia	< 5 years	Pneumonia hospitalizations	Mean hosp rate: <1yoa: 23.3% reduction 1-4yoa: 8.4% reduction	37,703 hospitalizations	30,101 hospitalizations
Schuck-Paim et al. 2019^32^	Nationwide	Ecological database study	Pre: 2004-2009 Post: 2010-2014	N/A	< 5 years	Pneumonia mortality		Between 1980 and 2010: national pneumonia mortality in children younger than 5 years decreased from about 150 to 15 deaths per 100 000 children younger than 5 years.	After 2010: vaccine-associated decline of about 10% in national childhood pneumonia mortality with our primary analytical method, with a high degree of uncertainty in the estimates. We observed larger reductions in municipal childhood pneumonia mortality in all three age groups (3–11 months, 3–23 months, and 3–59 months) in municipalities with a high percentage of extreme childhood poverty and mothers with no primary education, with the largest decrease observed in children aged 3–23 months in municipalities with low maternal education (24%, 95% credible interval 7–35).
**CAP**									
Silva et al. 2016^34^	Minas Gerais State	Ecological Database Study	2007 to 2013 (3 years)	5,044 cases (admissions)	< 1 year	CAP hospitalizations	Decline in prevalence: 19%	828 cases	624 cases Prevalence ratio: 0.81; 95%CI: 0.74 to 0.89; p<0.05
Sgambatti et al. 2015^33^	Goiania (Goias State)	Ecological Database Study	January to December 2012 (2 years)	3,353 records (both databases)	< 2 years	CAP			• SIH-SUS database: 5,285/1000,00 children, 95%CI 5,046 to 5,533) • APS database: 5,054/100,000 children, 95%CI 4,820 to 5,296
de Paulo Santana et al. 2017 (abstract)^39^	Rio de Janeiro State	Cross-sectional	Post: Jan 2015 to Sep 2016 (6 years)	63 patients	< 16 years	CAP	Descriptive: Greater number of hospitalizations among those < 6 months of age who were not vaccinated (p = 0.06) and that the hospitalization time was higher among those vaccinated (p = 0.007)		

**PM**									
Andrade et al. 2015 (abstract)^36^	Nationwide	Ecological Database Study	2008-2013 (3 years)	Not Stated	< 2 years	PMIPD	-44.4% (95%CI: -72.5; -15.8; p<0.002)		
Jarovsky et al. 2016^41^	Sao Paulo State	Surveillance	Pre: Jan 2000 - 2009 Post: 2010 - Dec 2015 (5 years)	98 eligible cases	All ages (stratified)	PM		Cases: 66 (6.6 cases/year)Median age:315.5 months (26 years)	Cases: 29 (4. 8 cases/year) Median age:76 months (6 years)
Azevedo et al. 2016^18^	Salvador (Bahia State)	Surveillance	Pre: Jan 2008 - May 2010Post: Jun 2010 - Dec 2012 (2 years)	148 cases of PM	All ages (stratified)	PM (NVT serotype isolates)		• Annual incidence (2008) - 0.9/100 000 • NVT: 0.69/100 000 • VT: 0.57/100 000	• Annual incidence (2012)- 0.36/100 000 (p<0.05) • NVT: 0.21/100 000 (P<0.76) • VT: 0.21/100 000 (P<1.0)
**AOM**									
Oliveira et al 2016^30^	Salvador (Bahia State)	Cohort	Sep 2009 and Oct 2013 (3 years)	576 children of which 422 followed-up	< 2 years	ARIAOM	PHiD-CV was inversely associated with AOM (OR [95%CI]: 0.16 [0.05 to 0.52])		
Sartori et al 2017b*^31^	Goiania (Goias State)	Ecological Database Study	Pre: Aug 2008 to Jul 2010Post: Aug 2011 to Jul 2015 (5 years)	6,401 OM outpatient visits were recorded in 4,793 children	< 2 years	All-case OM	Impact: 43.0% (95%CI 41.4% to 44.5%)		Visit reduction: 50.7%; 95%CI: 42.2% to 59.2%; p = 0.013
**Nasopharyngeal carriage**									
Brandileone et al. 2016^21^	Sao Paulo State	Cross-sectional	Pre: 2010Post: 2013 (3 years)	Overall 501 children (pre/baseline) and 1,167 (post) surveyed (including 400 tested for *Sp*)	< 2 years	VT *Sp* carriageVT NT*Hi* carriage	Effectiveness VT carriage: (3+1 schedule) 97.3% (95%CI: 88.7% to 99.3%)(2+1 schedule) 92.7% (95%CI: 79.6% to 97.4%) *Sp*: PHiD-CV serotypes were found in 19.8% and 1.8% respectively, representing a decline of 90.9% (p < 0.0001).	*Sp*: 40.3%NT*Hi*: 26.0%	*Sp*: 48.8%NT*Hi*: 43.6%
Neves et al. 2017^29^	Niteroi (Rio de Janeiro State)	Cross-sectional	Post: Sep and Dec 2014 (4 years)	522 children, 118 (22.6%) were pneumococcal carriers	< 6 years	Pneumococcal carriage			• < 2 years: 52/321 (16.2%) • ≥2 years of age: 66/201 (32.8%) colonized with *Sp* (p < 0.01).
Brandileone et al. 2019^22^	Sao Paulo State	Cross-sectional	Pre: 2010 Post: 2013, 2017	531 children	1 - 2 years	VT Spn carriage NVT Spn carriage	Spn carriage increased from 40.3% (baseline) to 59.7% (2017 survey) (p < 0.001). Colonization by VT isolates significantly decreased by 90.9% (19.8–1.8%) and 95.5% (19.8–0.9%) in the 2013 and 2017 surveys, respectively, compared to that at baseline. NVT isolates increased significantly by 128% (19.6–44.8%) and 185% (19.6–55.9%) in the respective post-PCV10 surveys, most led to high prevalence of serotypes 6C (27%), 15B (9.8%), 19A (9.2%), 15A (6.0%), and 16F (5.7%). In 2017, reduction in serotype 6A (4.2–0.6%, p < 0.001) and increase in serotype 19A (1.8–6.0%, p = 0.001) were found.		

*Included as abstract in previous article (Moreira et al, 2016).^3^

Abbreviations: AOM, acute otitis media; CAP, community acquired pneumonia; CI, confidence interval; IPD, invasive pneumococcal disease; NT*Hi*, non-typeable *Haemophilus influenzae*; NVT, non-vaccine type; PCV, pneumococcal conjugate vaccine; PHiD-CV, 10-valent pneumococcal and non-typeable *Haemophilus influenzae* protein D conjugate vaccine; PM, pneumococcal meningitis; *Sp, Streptococcus pneumoniae*; VT, vaccine-type.

Nine studies reported nationwide data,^17,19,20,23,25,30,32,36,38^ all other studies reported state or region-specific data (São Paulo [n = 8],^21,22,27,28,40–42,45^ Bahia [n = 3],^18,26,30^ Goiás [n = 2],^31,33^ Minas Gerais [n = 2],^34,40^ Rio de Janeiro [n = 2],^29,39^ Tocantins [n = 1],^43^ Santa Catarina [n = 2]^24,35^ and Maranhao [n = 1]^37^) (Figure 2A).

In terms of study designs, the most common was ecological database study (n = 13)^17,24,25,31–38,43,44^ followed by surveillance-based (hospital-based or laboratory-based) (n = 9),^18–20,23,28,40–42,45^ cross-sectional (n = 5),^21,22,27,29,39^ cohort (n = 1),^30^ and case series (n = 1) (Figure 2B).

The studies identified in this review assessed vaccine impact in specific age groups: <1-year-old (n = 3),^24,34,38^ <2 years (n = 7),^21,22,25,30,31,33,36^ 1–4 year-olds (n = 4),^32,35,37,43^ <6-years-olds (n = 3),^17,29,44^ and <16-years-olds (n = 3).^39,40,45^ Nine studies reported data in individuals of all ages (Figure 2C).^18–20,23,26–28,41,42^ Most studies reported IPD as the outcome (n = 10),^19,20,23,26–28,36,40,42,45^ followed by pneumonia (n = 9),^17,24,25,32,35,37,38,43,44^ community-acquired pneumonia (CAP) (n = 3),^33,34,39^ pneumococcal meningitis (n = 3),^18,21,41^ AOM/Otitis Media (OM) (n = 2)^30,31^ and nasopharyngeal carriage (n = 3).^21,22,29^ An overview of studies by outcome and target age group is provided in Figure 2D and Supplementary Figure 2.

Due to a high rate of heterogeneity (I2 = 96%) for all study outcomes, a meta-analysis of the extracted outcomes was not performed. This high rate of heterogeneity can be explained by the distinct types of study designs and multiple types of outcomes measured (i.e. clinical disease endpoints). Consequently, a descriptive overview of the individual studies is presented.

### PHiD-CV coverage and impact

#### Vaccination coverage

**Figure 3.**
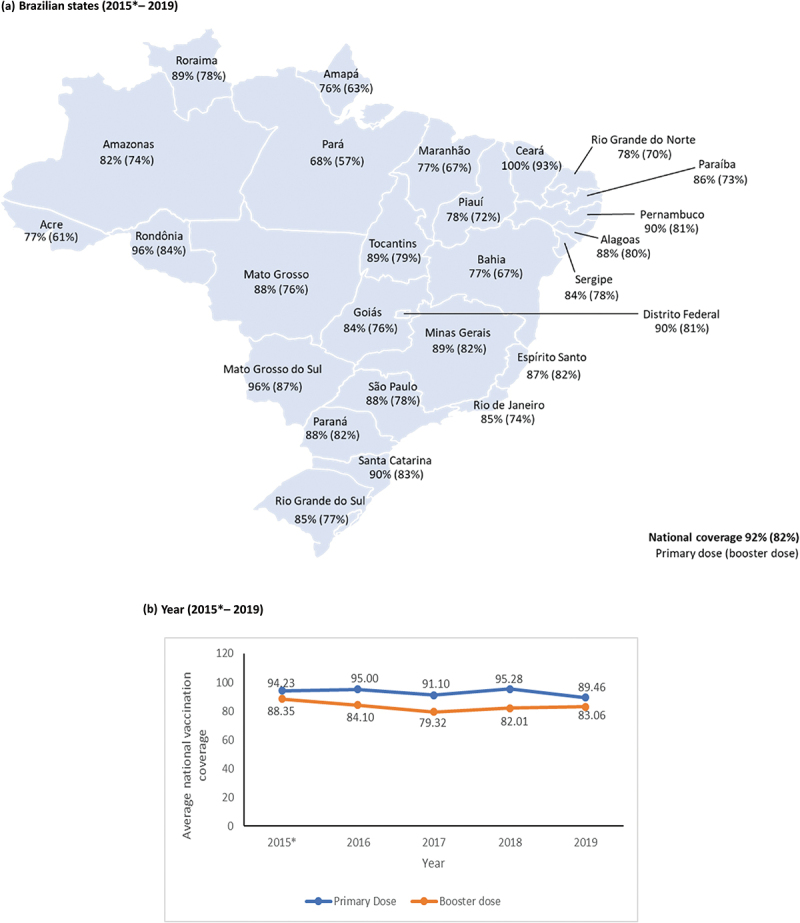
Average vaccination coverage of primary and booster doses of PHiD-CV per (A) Brazilian states (2015* – 2019) and (B) year Note: In 2015* there was a 3 + 1 schedule, and after 2016 there is a 2 + 1 schedule.

According to the Brazilian Ministry of Health administrative database (via DATASUS),^8^ the overall vaccination coverage is estimated with the last dose of each primary schedule. Vaccine coverage for both the primary schedule and the booster dose of PHiD-CV increased from 2010 until 2015 (3 + 1 schedule). However, from 2016 to 2019 (2 + 1 schedule), overall vaccine coverage decreased from 95% to 54.4% for the primary schedule. Coverage rates for the PHiD-CV booster dose also declined from 84.1% in 2016 to 50.5% in 2019. At the regional level, primary dose coverage decreased from 2016 to 2019 in all regions of the country: North (85.8% to 54.5%); Northeast (92.2% to 54.7%); South (96.7% to 59.8%); Southeast (96.9% to 51.9%) and Mid-West (100% to 56.9%). Booster dose coverage also declined in a comparable manner in all regions of the country. A similar decrease in primary dose coverage was noted among the different states in Brazil, with all states seeing a marked decline in coverage rates in 2019. Booster dose coverage in all states also declined from 2016 to 2019 (see Figure 3).

The health system estimates coverage of the complete schedule by proxy of the coverage of the last dose (in this case, 3rd dose until 2015 and 2nd dose since 2016).

#### Impact of PHiD-CV on IPD and pneumococcal meningitis

Evidence on the overall impact of PHiD-CV on IPD and pneumococcal meningitis was reported in ten studies,^19,20,23,26–28,36,40,42,45^ and three studies,^18,20,41^ respectively (Table 2). Overall, a decrease in the direct and indirect burden of IPD and pneumococcal meningitis after PHiD-CV introduction was documented in children < 5 years of age, with a higher number of cases of pneumococcal meningitis reported in adults. While there are reports of increasing numbers of IPD and meningitis cases linked to non-vaccine serotypes in children below five years, a normal fluctuation of prevailing serotypes over the years has also been documented.

An interrupted time series analysis conducted nationwide between 2008 and 2013 indicated that PHiD-CV significantly reduced IPD and pneumococcal meningitis rates by 44.4% (95%CI: −72.5 to −15.8; *p* <.002) in children 2 months to <2 years of age.^36^ Evidence from a retrospective hospital-based surveillance study showed a lower proportion of IPD cases among younger children between the pre-vaccination and post-vaccination periods: <2 years: 60% and 42%; 2–5 years: 23% and 28%; >5 years: 16% and 25%).^40^ In an observational study conducted in Salvador municipality between 2010 and 2013, 82 IPD cases were included, from which 14 were reported in children under 2 years of age, and 3 (21.4%) were vaccinated prior to the IPD episode.^26^

Another nationwide hospital-based surveillance study reported a decline in hospitalizations from 20 cases to 5 cases and deaths from 6.6 to 2.0 cases per 10,000 pediatric admissions when comparing pre- and post-vaccine introduction periods.^19^ Overall 30.0% of cases were sent to the ICU regardless of vaccine introduction.^19^

A retrospective cross-sectional study analyzed records of 332 isolated pneumococcal strains in patients of all age groups (168 isolates for <5 year-olds) with IPD between 1998 and 2013 in the University Hospital in Ribeirão Preto municipality, São Paulo.^27^ The study showed a decrease from 61.7% (2003–2010) to 13.9% (2011–2013) in the number of IPD cases.^27^ A hospital-based surveillance study conducted in Salvador municipality (Bahia State) that compared the incidence of pneumococcal meningitis before (January 2008-May 2010) and after (June 2010-December 2012) PHiD-CV introduction estimated a reduction in the annual incidence of pneumococcal meningitis (0.9 per 100,000 inhabitants in 2008 to 0.36 per 100,000 inhabitants in 2012; *P* <.05). However, while the incidence of both vaccine-type pneumococcal meningitis (from 0.57 per 100,000 inhabitants in 2010 to 0.21 per 100,000 inhabitants in 2012; *P* <1.0) and non-vaccine-type pneumococcal meningitis (from 0.69 per 100,000 inhabitants in 2008 to 0.21 per 100,000 inhabitants in 2012; *P* <.76) declined, the reductions were not significant. Of note, during the initial period after PHiD-CV use, there was no emergence of a specific serotype among non-PHiD-CV strains. A comparison of the number of 12 F, 10A, 15B and 18B serotype isolates identified before and after PHiD-CV vaccine introduction suggested a normal fluctuation of these serotypes over the years, with no evidence of specific emergence of any particular serotype. It is important to consider that in this study the majority of cases were reported in adults (63.4%), who are not the target group for vaccination. During the study period, there was no evidence demonstrating the effect of eventual replacement of the PHiD-CV serotypes.^18^

In a retrospective follow-up study that analyzed 796 isolates in individuals of all ages in the Northeast region of São Paulo State, the number of IPD cases dropped from 60 cases per year from 2003 to 2010 to 32 cases per year in 2011–2013; notably, the cases due to serotypes 14, 1, 23 F and 5 had declined.^28^ In addition, a national laboratory-based surveillance study from 2005 to 2015 that analyzed 8,971 IPD isolates from patients across age groups of 2 months to 99 years showed a reduction in vaccine-type IPD meningitis cases, ranging from 83.4% in 2 months to 4 years age group to 47.7% in >65 years age group (non-vaccine target) 5-years post-vaccine introduction in Brazil.^20^ Three hospital-based surveillance studies in São Paulo City showed a reduction in the overall cases of IPD including meningitis in Brazil post-introduction of PHiD-CV.^41,42,45^ The number of pneumococcal meningitis cases decreased from 7 cases per year (for pre-vaccination period: 2000–2009) to 5 cases per year (for post-vaccination period: 2010–2015).^41^ In a prospective hospital-based surveillance study of patients with IPD from January 2000 to April 2017, an annual decrease of 15.6% in the number of IPD cases in all age groups was observed from 2010 to 2017 without evidence of serotype replacement. Notably, in healthy individuals, a 57.3% reduction in the number of IPD cases was reported.^42^ In a hospital-based surveillance study, the number of IPD cases among individuals <15 years of age declined from 25 cases per year from 2005 to 2010 to 9 cases per year from 2011 to 2016.^45^

#### Impact of PHiD-CV on all-cause pneumonia and CAP

Nine studies evaluated and reported the impact of PHiD-CV introduction on pneumonia (Table 2).^17,24,25,32,35,37,38,43,44^ Five studies evaluated the impact of vaccination with PHiD-CV on pneumonia-related mortality,^24,32,38,43,44^ while five studies evaluated the impact of vaccination with PHiD-CV on pneumonia hospitalizations.^17,25,35,37,43^ Overall, decrease in pneumonia-related mortality at both national and state level was documented after the introduction of PHiD-CV vaccination. Similarly, a decrease in the number or proportion of pneumonia hospitalizations post-PHiD-CV introduction was recorded throughout the country. One a few studies assessed the impact of PHiD-CV vaccination on CAP hospitalizations; a decline in CAP hospitalization was documented in the post-vaccine era.

In one ecological study in Santa Catarina State that analyzed data from the Mortality Information System of children <1 year of age, a reduction of 11% deaths in all-cause pneumonia-related deaths was reported (from 29.7 to 23.4 per 100,000 between 2006–2009 and 2010–2013, respectively).^24^ In a retrospective analysis conducted in children 1–4 years of age in the Tocantins State, a 28% (significant) reduction in pneumonia-related deaths were observed two years post-vaccine introduction in Brazil (2011–2013) compared to the pre-vaccination period (2008–2010).^43^ Another study reported a reduction of 21% in mortality among children <5 years of age (from 0.746 death per 1,000 live births to 0.589 death per 1,000 live births) between 2006–10 and 2011–15, respectively.^44^ In a nationwide retrospective observational study (with a 34-year time-series analysis) of children <5 years of age, a 10% reduction in pneumonia-related mortality was estimated post-vaccination; however, the analytical methods used showed a high degree of uncertainty to generalize these conclusions.^32^

A decline in all-cause pneumonia hospitalizations was also documented in Brazil several years after PHiD-CV introduction in Brazil. One study (2003–2013) reported a nationwide reduction after 48 months of vaccine introduction of 10% (95%CI: 4% to 19%), 7% (95%CI: 1% to 10%) and 11% (95%CI: 4% to 13%) in all-cause pneumonia hospitalizations in infants <12 months of age, children 12–23 months of age and children 24–59 months of age, respectively.^25^ A retrospective analysis conducted in Maranhao State estimated a 10% (not significant) reduction in all-cause pneumonia hospitalizations in children 1–4 years of age after PHiD-CV introduction (2011–2013) compared to the pre-vaccination period (2008–2010).^37^ In another retrospective analysis conducted in children 1–4 years of age in the Tocantins State, a 9% reduction in pneumonia hospitalizations was observed post-vaccine introduction in Brazil (2011–2013) compared to the pre-vaccination period (2008–2010).^43^ Comparing pneumonia hospitalization rates pre- and post-vaccination years between 2005 and 2015 (i.e. 5-year post-vaccine introduction) in children <5 years of age, a significant impact of vaccination was observed in infants <12 months of age (−26.5% [95%CI: −35.5% to −17.5%]; *p* = .001). During the same time period, a significant decrease in pneumonia hospitalization rate was observed in children 2–4 years of age, with a relative percentage of −21.5% (95%CI: −29.8% to −13.2%; *p* = .002).^17^ An ecological database study conducted in the Santa Catarina state among children <5 years of age reported that pneumonia-related hospitalizations reduced from 37,703 (pre-vaccine introduction) to 30,101 (post-vaccine introduction).^35^ The study also reported that the mean hospitalization rate declined by 23.3% for children <1 years of age and 8.4% for children 1–4 years of age.^35^

Three studies described the impact of PHiD-CV introduction on CAP (Table 2).^33,34,39^ In a time series analysis conducted in a selected region of Minas Gerais state from 2007 to 2013, a significant 19% reduction in CAP hospitalization rate was observed in infants <1 year of age with the number of hospitalized cases dropping from 828 to 624 three years after vaccine introduction (prevalence ratio: 0.81; 95%CI: 0.74 to 0.89; *p* <.05).^34^ In a retrospective cross-sectional study conducted in Rio de Janeiro city from January 2015 to September 2016, the number of hospitalizations was higher among unvaccinated infants <6 months of age compared to vaccinated infants (*p* = .06).^39^

#### Impact of PHiD-CV on AOM

Overall there was a positive impact of PHiD-CV introduction on the burden of AOM^30,31^ (Table 2). A prospective cohort study conducted in Salvador municipality (2009 and 2013) showed that in vaccinated children 6–23 months of age who were diagnosed with acute respiratory infection during 2010 and 2013, PHiD-CV immunization was associated with low odds of developing AOM (odds ratio [OR]: 0.16 [95%CI: 0.05 to 0.52]).^30^ In an interrupted time-series analysis, a significant impact of PHiD-CV immunization in children 2–23 months of age was measured using the data captured in Goiania electronic Outpatient Visit Information System (OVIS) from August 2008 to July 2015. Five years after vaccine introduction, a significant reduction in all-cause OM visits was observed and the impact of PHiD-CV on all-cause OM was estimated at 43.0% (95%CI: 41.4% to 44.5%).^31^

#### Nasopharyngeal carriage

Three studies evaluated the effect of PHiD-CV on nasopharyngeal carriage (Table 2).^21,22,29^ Overall, nasopharyngeal carriage of vaccine-type *Streptococcus pneumoniae* decreased in children, with the highest decline observed in children <2 years of age. Vaccine-related serotypes 6A and 19A did not increase, yet carriage of other non-vaccine type isolates increased and an increase in antimicrobial resistance was also reported; this was specific to the emergence of serotype 6 C and 19A isolates.^21,29^

One study detected *Streptococcus pneumoniae* in 40.3% of children 12–23 months at baseline (2010) and in 48.8% of children post-vaccine introduction. In this study, vaccine serotypes were found in 19.8% and 1.8% of children at baseline and post-vaccine introduction, respectively, representing a decline in nasopharyngeal carriage of 90.9% (*p* <.0001). Vaccine effectiveness of the 3 + 1 and 2 + 1 dose schedule against vaccine-type carriage was 97.3% (95%CI: 88.7% to 99.3%) and 92.7% (95%CI: 79.6% to 97.4%), respectively.^21^ In another cross-sectional study conducted in Niteroi city (Rio de Janeiro) four years after vaccine introduction, pneumococcal nasopharyngeal carriage was evaluated in children ≤6 years of age. A lower prevalence of *Streptococcus pneumoniae* colonization was observed in children <2 years of age (16.2%; *p* <.01) compared to children ≥2 years of age (32.8%; *p* <.01).^29^ Among children ≥2 years of age, the highest prevalence of colonization was observed among children of 2–4 years of age (36%).^29^ Serotypes 6 C, 15B/C, 11A/D, 6A, 15A/F, 23A, and 23 were the most common capsular types observed in this study. Resistance to several antimicrobial agents was frequently observed and this was mainly associated with the emergence of multidrug-resistant serotype 6 C.^29^ In another cross-sectional study from Sao Paulo state in children 1–2 years of age, *Streptococcus pneumoniae* carriage increased from a baseline of 40.3% in 2010 to 59.7% in 2017, and vaccine-type isolates significantly decreased by 90.9% and 95.5% in 2013 and 2017, respectively.^22^ The study also reported that non-vaccine-type isolates increased significantly by 128.0% and 185.0% in 2013 and 2017, respectively.

#### Risk of bias and quality assessment

Risk of bias was performed for the full-text journal publications (n = 19) using the STROBE checklist. As presented in Supplementary Figure 3A, the overall risk of bias was high in 11 studies and moderate in 8 studies. The high overall risk of bias of individual studies was driven mainly by the choice of methods for selecting study participants (lack of an appropriate population and pre-defined criteria for study eligibility) and a lack of methods to control confounding. In addition, the overall risk of bias was high due to a high design-specific source of bias which can be attributed to the nature of observational studies, specifically those using passive surveillance and laboratory data.

The quality of evidence and strength of recommendation was evaluated for the full-text journal publications using GRADE (Supplementary Figure 3B).^46^ The varying quality of evidence reflected that study designs of the included studies had various limitations. Study heterogeneity, lack of generalizability of the study findings beyond the studied population and imprecision were identified as reasons for the low quality of evidence. Therefore, the strength of recommendation evaluation categorized studies into weak (n = 10), medium (n = 6) and strong (n = 3) for further recommendation. In the context of the main findings of this study, it is reasonable to infer that PHiD-CV had a positive direct and indirect impact on pneumococcal disease in children <5 years of age and conferred an adequate herd effect in the unvaccinated population.

## Discussion

In this paper, we present an up-to-date review of the evidence on the impact of the routine immunization program with PHiD-CV in Brazil, particularly following the switch from a 3 + 1 to a 2 + 1 schedule in 2016. A descriptive analysis was presented instead of performing a meta-analysis due to the high heterogeneity between studies. The review identified a total of 29 studies (19 publications and 10 abstracts). Nine studies were nationwide studies and were considered representative of the national population,^17,19,20,23,25,32,36,38,44^ while the remaining studies were conducted in one or several regions. The review reveals that the burden of IPD, all-cause pneumonia, and AOM has considerably decreased since the introduction of PHiD-CV in Brazil, with heterogeneous effects across states. The impact of PHiD-CV in the general population of Brazil are consistent with the findings from other countries including those in the Latin America and Caribbean region.^47–50^

Findings from this review support the results of the original review that the PHiD-CV program in Brazil has a substantial direct and indirect positive impact on the IPD and noninvasive disease burden caused by vaccine-types, and furthermore evaluates the impact during the post-vaccination period after the change in vaccination scheme.^3^ Among the vaccine target population, particularly < 5 year olds, vaccine-type IPD declined substantially post PHiD-CV introduction.^20,26–28,36^ Six studies reported that vaccination with PHiD-CV conferred a substantial reduction in vaccine-type IPD in the population not targeted by vaccination, in particular after 3 years of PHiD-CV use, leading to broader protection against vaccine-type IPD in all age groups.^17,20,27,28,42,45^ It is known that pneumonia and AOM, when compared to IPD poses a higher overall public health burden and management costs from a societal perspective.^16^ Importantly, studies from Brazil assessing the effectiveness of PHiD-CV have reported a reduction in pneumonia mortality and hospitalizations,^17,24,32,35,37,38,43,44^ and evidence of indirect (herd) protection in unvaccinated older children and adults in highly vaccinated populations.^17,37,43^ One study showed a relative reduction in pneumonia hospitalization in the target vaccine group with an indirect positive effect in individuals 10–49 years of age.^17^ This review also identified revealed a significant reduction in all-cause OM disease in young children, with rates of all-cause OM visits significantly reduced since the introduction of PHiD-CV vaccination in Brazil.^31^ OM is one of the main reasons for seeking healthcare services and antibiotic prescription during childhood and therefore this reduction is reflective of the public health impact of the vaccination program in Brazil.

Published literature points to a shift in the serotype distribution seven years after the introduction of PHiD-CV in Brazil; an increase in non-vaccine serotypes was reported as a cause of disease in both children and older adults.^20,23,26–28,40^ One study reported an increase in serotype 19A among children <5 years (attributed mainly to emergence multidrug-resistant strains)^23^ and another study reported increasing levels of carriage of non-vaccine types.^22^ Yet, other studies did not show serotype replacement among children <2 and <5 years of age,^18,26,42^ demonstrating the real value of PCV in keeping the net effect. Agudelo et al. conclude that in Latin America, there has been an overall reduction in the trend and number of invasive *Streptococcus pneumoniae* isolates in children <5 years after PCVs introduction. However, the prevalence of specific serotypes, such as 19A, has a relatively increasing trend, which is observed and sustains in most countries regardless of the vaccine used.^50^ The increase in non-vaccine-type IPD after PHiD-CV introduction might be related to several factors such as antibiotic over-usage, pneumococcal carriage, the prevalence of IPD underlying illness or comorbidities, heterogeneity in vaccine coverage levels across a nation and the use of a passive laboratory-based surveillance system.^51^

Non-vaccine types are now becoming an important cause of IPD regardless of differences in PCV programs (such as previous use of the 7-valent PCV, choice of higher valent PCV or immunization schedules), despite epidemiological differences (such as pre-PCV incidence or serotype distribution) and vaccination program (with catch-up scheme).^52^

Globally, it is not clear whether any serotype plays a more prominent role in causing disease in countries using one PCV vaccine over the other.^53,54^ In a recent review that assessed the relative importance of non-vaccine types after higher valent PCV implementation, regardless of the PCV program used, there is an increase in all IPD cases caused by non-vaccine types in distinct age groups.^55^ Focusing on the increase in serotype 3 circulation, it remains unknown whether this is due to the vaccine effect of PCV-13, or replacement by other non-vaccine types in the PHiD-CV countries (and therefore proportionately higher levels of serotype 3).^55,56^ An increase in serotype 3 circulation may also be independent of the specific vaccine and related with vaccine coverage or previous differences in serotype distribution. Overall, this situation demands careful monitoring, and factors influencing the increase in non-vaccine type disease needs to be systematically investigated. Importantly, efforts should be made to improve national surveillance systems (for example, moving away from passive to active surveillance for the most prevalent invasive diseases in Brazil) so that any heterogeneity between studies that confounds interpretation of PCV impact data can be minimized at the outset. Equally important is the continuous surveillance in individuals of all ages based on standardized methods to ascertain the long-term effects of PCV immunization programs in terms of serotype replacement.^50^

Only few longitudinal (i.e. > 20 years) post-vaccination studies have estimated the impact of PCV use on childhood mortality. For example, a recent study with time-series analysis of 27 years (20 years pre- and 7 years post-vaccination) observed continuous annual reduction in mortality from lower respiratory infections (as a proxy to pneumonia) and pneumococcal meningitis among Brazilian children <5 years of age, irrespective of vaccination.^57^

Colonization of the nasopharynx is acknowledged as a precursor for pneumococcal disease and is required for the transmission of the pneumococcus to other individuals. A vaccine that reduces colonization by vaccine serotypes could be expected to both decrease the risk of disease in the vaccinated individual and provide herd protection to unvaccinated individuals. On the other hand, increases in non-vaccine serotype colonization as a result of vaccination referred to as “replacement” carriage, have the potential to lead to increased non-vaccine disease in both vaccinated and unvaccinated populations.^58^ In this review, a decrease in nasopharyngeal carriage of vaccine-type and any-type pneumococci was reported in two studies.^21,29^ Serotypes 6 C, 15B/C, 11A/D, 6A, 15A/F, 23A, and 23 were the most common capsular types observed in Brazil.^21^ Vaccine effectiveness of the 3 + 1 and 2 + 1 dose schedule against vaccine-type carriage was 97.3% and 92.7%, respectively.^21^ Over time, the PHiD-CV 2 + 1 schedule may have a large impact on the carriage. Given the recent shift in the vaccination schedule in Brazil, further monitoring of the impact of the vaccine in the nasopharyngeal carriage is warranted to elucidate the population-level impact of PHiD-CV in Brazil.

Overall, the findings from this review can be corroborated by the national coverage rates in Brazil which increased until 2015. Since 2016, the vaccination coverage rate of the PHiD-CV has declined in Brazil.^8^ Notably, this decline has been documented for other vaccines; one can note the high homogeneity in the first decade of the 2000s (coverage rates of ≥95%) and a drop since 2014. Low vaccination coverage rates could compromise any vaccination program, independently of the type of PCV used. Reasons for decline in coverage have not been explicitly stated by the Brazilian Ministry of Health but recent published literature report a multitude of contributing factors that could be related such as changes in the national immunization information system,^59^ misinformation about the importance of vaccines and administration schedule and vaccine hesitancy (i.e. delay in acceptance or refusal despite having the recommended vaccines available in health services).^60^ These factors are the most commonly cited reasons for declining coverage in Brazil,^61,62^ and vaccine hesitancy has been reported as a main concern for Brazilian public administrators and researchers.^62^ In addition, timeliness of vaccination is an issue in Brazil. Evidence from a population-based cohort study conducted in 2012 shows that PHiD-CV vaccination (3 + 1 schedule) in children was delayed by 9.4%, 23.8%, 36.8% and 39.9% for the first, second, third and the booster doses, respectively.^63^ These findings cause concern since Brazil has recently switched to a new 2 + 1 PHiD-CV schedule,^4^ and the higher incidence of IPD is in the second half of the first year of life.

Several limitations must be considered in the interpretation of the overall findings. Most studies report a limited number of outcomes to assess vaccine effectiveness or impact. For instance, few studies reported the impact of vaccine introduction on vaccine-type IPD and others reported the impact on overall IPD. This could potentially limit the extrapolation of the main observations of this review. Overall, most of the studies were observational and surveillance-based which utilized secondary data sources such as hospitalizations or laboratory databases; with unclear indication of the method used for pneumococcal disease diagnosis (i.e. radiology, molecular, etc.) or case-definition. Data from surveillance studies are based on passive reporting by clinicians or other healthcare providers. Passive surveillance covers wide areas (whole regions or states); however, because it relies on an extensive network of healthcare providers, it becomes difficult to ensure completeness and timeliness of data. In addition, most of the studies were non-comparative and therefore any results need to be carefully interpreted as this setup tends to pose limitations in terms of bias and confounding.

Also, this review mostly included ecological database studies with time-series analysis which inherently placed an ecological bias on some interpretations; mostly due to the short/medium-term (i.e. < 8 years post-vaccination) time periods that were included in the analysis. To address this, longer-term data from surveillance or prospective studies are needed to evaluate longitudinal trends in time-series analysis.^57^

Furthermore, a lack of the consistency of age groups reported in the studies prevented direct comparisons between them. Similarly, the pre- and post-vaccination periods considered in the studies were inconsistent and therefore the overall findings should be interpreted in the context of the study design, the target population, and the setting in which the study was conducted. In terms of the design of the review, there is a potential for publication bias, although we tried to mitigate this by using a comprehensive list of data sources, a sensitive search strategy, and selection of papers using predefined eligibility criteria.

## Conclusion

In summary, the findings from this review confirm that the positive impact of PHiD-CV after 10 years of use in Brazil has sustained reductions in the disease burden of IPD, pneumonia and AOM, reducing the levels of nasopharyngeal carriage and providing indirect protection. Given the relatively recent implementation of the 2 + 1 PHiD-CV schedule in 2016, continuous surveillance is essential to evaluate the sustainability of the positive impact of PHiD-CV to assess changing serotype-specific disease trends are with a focus on transmission dynamics, population risk factors and pathogen evaluation. More importantly, there is an urgent need to address the declining pneumococcal vaccination coverage in Brazil.

## Supplementary Material

Supplemental Material

## Trademark

Synflorix is a trademark of GlaxoSmithKline Vaccines.
