# Supplementary material for: Impact after 10-year use of pneumococcal conjugate vaccine in the Brazilian national immunization program: an updated systematic literature review from 2015 to 2020
Source: Hum Vaccin Immunother. 2021 Mar 18;18(1):1879578. doi: 10.1080/21645515.2021.1879578 (PMC8920160; doi:10.1080/21645515.2021.1879578)
Supplement: Supplemental Material [file KHVI_A_1879578_SM9885.docx]

# Supplementary material

**Supplementary Table 1. Keywords used in search strategy (for title and abstract)**

| **Main Terms [OR]** | **Boolean Operator** | **Combined Terms [OR]** | |
| --- | --- | --- | --- |
|  |  |  |  |
| **Brasil OR Brazil** | **AND** | **Specific terms related to:** Vaccines Otitis Pneumonia Invasive disease Carriage Herd protection Other related diseases or health problems | |
| **AND** |  |  |  |
| **2015-2020** |  |  |  |

**Supplementary Table 2. Search strategy for PubMed (via Medline)**

| General String | (Brazil*[tw] OR Brasil*[tw]) AND ("2018/05/01"[Date - Publication] : "2020/05/1"[Date - Publication]) AND ("english"[Language] OR "portuguese"[Language] OR "spanish"[Language]) |
| --- | --- |
| Vaccines | "Pneumococcal Vaccines"[Mesh] OR pneumococcal conjugate vaccin*[tw] OR 10-valent[all] OR PHiD-CV[all] OR PCV10[all] OR PCV-10[all] OR Synflorix[all] OR ten-valent[all] OR pneumococcal non-typeable Haemophilus influenzae protein D conjugate vaccine |
| Otitis | (“Otitis media”[Mesh] OR otitis[tw] OR acute otitis media[tw] OR AOM[tw] OR OM[tw] OR OME[tw] OR tympanostomy[tw] OR myringotomy[tw] OR otorrhea[tw] OR chronic suppurative otitis media [tw] OR CSOM[tw]) AND (epidemiology[tw] OR prevalence[tw] OR incidence[tw]) |
| Pneumonia | ("Pneumonia"[Mesh] OR “Community-Acquired Infections”[Mesh] OR CAP[tw] OR lower respiratory tract infection*[tw] OR respiratory infection[tw]) AND (epidemiology[tw] OR prevalence[tw] OR incidence[tw]) |
| Invasive Disease | (“Invasive Pneumococcal Disease“[tw] OR IPD[tw] OR invasive bacterial disease[tw] OR “Bacteremia”[Mesh] OR bacteremi*[tw] OR bacteraemi*[tw] OR “Meningitis”[Mesh] OR sepsis[tw] or empyema[tw] OR septicemia[tw] OR septicaemia[tw]) AND (epidemiology[tw] OR prevalence[tw] OR incidence[tw])  AND (Respiratory bacterial pathogen*[tw] OR "Streptococcus pneumoniae"[Mesh] OR streptococcus pneumonia*[tw] OR S. pneumonia*[tw] OR pneumococcal[tw] OR pneumococ*[tw] OR "Haemophilus influenzae"[Mesh] OR haemophilus influenza*[tw] OR NTHi[tw] OR Hib[tw] OR hemophilus[tw] OR haemophilus[tw]) |
| Carriage | ("Carrier State"[Mesh] OR carriage[tw] OR "Nose/microbiology"[Mesh] OR "pharynx/microbiology"[Mesh] OR "Nasopharynx/microbiology"[Mesh] OR "Oropharynx/microbiology"[Mesh] OR colonization[tiab] OR colonisation[tiab] OR colonizing[tiab] OR colonising[tiab] OR bacterial load[tiab] OR bacterial density[tiab]) AND (epidemiology[tw] OR prevalence[tw] OR incidence[tw])  AND (Respiratory bacterial pathogen*[tw] OR "Streptococcus pneumoniae"[Mesh] OR streptococcus pneumonia*[tw] OR S. pneumonia*[tw] OR pneumococcal[tw] OR pneumococ*[tw] OR "Haemophilus influenzae"[Mesh] OR haemophilus influenza*[tw] OR NTHi[tw] OR Hib[tw] OR hemophilus[tw] OR haemophilus[tw]) |
| Herd Protection | ("Immunity, Herd”[Mesh] OR herd protection[tw] OR herd immunity[tw] OR indirect protection[tw] OR indirect immunity[tw] OR herd effect[tw]) AND (Respiratory bacterial pathogen*[tw] OR "Streptococcus pneumoniae"[Mesh] OR streptococcus pneumonia*[tw] OR S. pneumonia*[tw] OR pneumococcal[tw] OR pneumococ*[tw] OR "Haemophilus influenzae"[Mesh] OR haemophilus influenza*[tw] OR NTHi[tw] OR Hib[tw] OR hemophilus[tw] OR haemophilus[tw]) |
| Other related diseases | (bronchitis[tw] OR bronchiolitis[tw] OR asthma[tw] OR reactive airway disease[tw] OR sinusitis[tw] OR pharyngitis[tw] OR tonsillitis[tw] OR conjunctivitis[tw])  AND (epidemiology[tw] OR prevalence[tw] OR incidence[tw])  AND (child*[tw] OR pediatric*[tw] OR paediatric*[tw]) |

**Supplementary Table 3. Search strategy for Web of Science**

| General String | TITLE: (brazil OR brasil OR Brasil OR Brazil OR "brasil" OR "brazil") Timespan: 2018-2020. Indexes: SCI-EXPANDED, SSCI, A&HCI, CPCI-S, CPCI-SSH, BKCI-S, BKCI-SSH, ESCI, CCR-EXPANDED, IC. |
| --- | --- |
| Vaccines | TITLE: (vaccine OR pneumococcal OR Pneumococcal Vaccines OR pneumococcal conjugate vaccine OR 10-valent OR PHiD-CV OR PCV10 OR PCV-10 OR Synflorix OR ten-valent OR pneumococcal non-typeable Haemophilus influenzae protein D conjugate vaccine) AND (epidemiology OR prevalence OR incidence) |
| Otitis | TITLE: (Otitis media OR otitis OR acute otitis media OR AOM OR OM OR OME OR tympanostomy OR myringotomy OR otorrhea OR chronic suppurative otitis media OR CSOM)  AND (epidemiology OR prevalence OR incidence) |
| Pneumonia | TITLE: (Pneumonia OR Community-Acquired Infections OR CAP OR lower respiratory tract infection OR respiratory infection)  AND (epidemiology OR prevalence OR incidence) |
| Invasive Disease | TITLE: (Invasive Pneumococcal Disease OR IPD OR invasive bacterial disease OR Bacteremia OR bacteremia OR bacteraemia OR Meningitis OR sepsis or empyema OR septicemia OR septicaemia) AND (Respiratory bacterial pathogen OR "Streptococcus pneumoniae" OR streptococcus pneumonia OR S. pneumonia OR pneumococcal OR "Haemophilus influenzae" OR haemophilus influenza OR NTHi OR Hib OR hemophilus OR haemophilus OR pneumococ) AND (epidemiology OR prevalence OR incidence) |
| Carriage | TITLE: (Carrier State OR carriage OR Nose microbiology OR pharynx microbiology OR Nasopharynx microbiology OR Oropharynx microbiology OR colonization OR colonisation OR colonizing OR colonising OR bacterial load OR bacterial density) AND (Respiratory bacterial pathogen OR "Streptococcus pneumoniae" OR streptococcus pneumonia OR S. pneumonia OR pneumococcal OR "Haemophilus influenzae" OR haemophilus influenza OR NTHi OR Hib OR hemophilus OR haemophilus OR pneumococ) AND (epidemiology OR prevalence OR incidence) |
| Herd Protection | TITLE: (herd protection OR herd immunity OR indirect protection OR indirect immunity OR herd effect)  AND (Respiratory bacterial pathogen OR "Streptococcus pneumoniae" OR streptococcus pneumonia OR S. pneumonia OR pneumococcal OR "Haemophilus influenzae" OR haemophilus influenza OR NTHi OR Hib OR hemophilus OR haemophilus OR pneumococ) |
| Other related diseases | TITLE: (bronchitis OR bronchiolitis OR asthma OR reactive airway disease OR sinusitis OR pharyngitis OR tonsillitis OR conjunctivitis) AND (child OR children OR pediatric OR paediatric) AND (epidemiology OR prevalence OR incidence) |

**Supplementary Table 4. Search strategy for Scopus**

| General String | ( TITLE-ABS-KEY ( brazil OR brasil OR Brasil OR Brazil OR "brasil" OR "brazil" ) ) AND ( LIMIT-TO ( LANGUAGE , "English" ) OR LIMIT-TO ( LANGUAGE , "Portuguese" ) ) AND ( LIMIT-TO ( PUBYEAR , 2018 ) OR LIMIT-TO ( PUBYEAR , 2019 ) OR LIMIT-TO ( PUBYEAR , 2020 ) ) AND ( TITLE-ABS-KEY ( "synflorix" OR "pneumococcal conjugate vaccine" OR pcv10 OR "pcv 10" OR "10 valent" ) ) |
| --- | --- |
| Vaccines | AND ( TITLE-ABS-KEY ( "synflorix" OR "pneumococcal conjugate vaccine" OR pcv10 OR "pcv 10" OR "10 valent" ) ) |
| Otitis | AND ( TITLE-ABS-KEY ( otitis OR media OR aom OR tympanostomy OR “myringotomy” OR “otorrhea” OR chronic OR suppurative ) ) AND ( TITLE-ABS-KEY ( “epidemiology” OR “prevalence” OR “incidence”)) |
| Pneumonia | AND ( TITLE-ABS-KEY ( “pneumonia” OR “community acquired” OR “lower respiratory tract infection” OR “respiratory infection”)) AND ( TITLE-ABS-KEY ( “epidemiology” OR “prevalence” OR “incidence”)) |
| Invasive Disease | AND ( TITLE-ABS-KEY ( “invasive and pneumococcal and disease” OR ipd OR “invasive disease” OR “bacteremia” OR “meningitis” OR “sepsis” OR “septicaemia” OR “streptococcus “pneumoniae OR pneumococcal OR pneumococcus OR “haemophilus” OR influenzae OR nthi OR hib OR “respiratory bacterial pathogen”)) AND ( TITLE-ABS-KEY ( “epidemiology” OR “prevalence” OR “incidence”)) |
| Carriage | AND ( TITLE-ABS-KEY ( carrier OR carriage OR “nose” OR “pharynx” OR “nasopharynx” OR “oropharynx” OR “microbiology” OR colonization OR colonisation OR colonizing OR colonising OR “bacterial load” OR “bacterial density”) AND (“streptococcus” OR pneumoniae OR pneumococcal OR pneumococcus OR “haemophilus influenzae” OR nthi OR hib OR “respiratory bacterial pathogen”) AND ( TITLE-ABS-KEY ( “epidemiology” OR “prevalence” OR “incidence”)) |
| Herd Protection | AND ( TITLE-ABS-KEY (“herd immunity” OR “herd protection” OR “herd effect” OR “indirect protection” OR “indirect immunity” OR “indirect effect”)) AND ( TITLE-ABS-KEY (“streptococcus” OR pneumoniae OR pneumococcal OR pneumococcus OR “haemophilus influenzae” OR nthi OR hib OR “respiratory bacterial pathogen”))) |
| Other related diseases | AND ( TITLE-ABS-KEY (“bronchitis” OR “bronchiolitis” OR “asthma” OR “reactive airway disease” OR “sinusitis” OR “pharyngitis” OR “tonsillitis” OR “conjunctivitis”) AND (“child” OR pediatric OR paediatric) AND ( TITLE-ABS-KEY ( “epidemiology” OR “prevalence” OR “incidence”)) |

**Supplementary Table 5. Search strategy for LILACS**

| General String | (brazil OR brasil OR Brasil OR Brazil OR "brasil" OR "brazil") [Words] and 2018 OR 2019 OR 2020 [Country, year publication] |
| --- | --- |
| Vaccines | (vaccine OR pneumococcal OR Pneumococcal Vaccines OR pneumococcal conjugate vaccine OR 10-valent OR PHiD-CV OR PCV10 OR PCV-10 OR Synflorix OR ten-valent OR pneumococcal non-typeable Haemophilus influenzae protein D conjugate vaccine) [Words] |
| Otitis | (Otitis media OR otitis OR acute otitis media OR AOM OR OM OR OME OR tympanostomy OR myringotomy OR otorrhea OR chronic suppurative otitis media OR CSOM)  AND (epidemiology OR prevalence OR incidence) [Words] |
| Pneumonia | (Pneumonia OR Community-Acquired Infections OR CAP OR lower respiratory tract infection OR respiratory infection)  AND (epidemiology OR prevalence OR incidence) [Words] |
| Invasive Disease | (Invasive Pneumococcal Disease OR IPD OR invasive bacterial disease OR Bacteremia OR bacteremia OR bacteraemia OR Meningitis OR sepsis or empyema OR septicemia OR septicaemia) AND (Respiratory bacterial pathogen OR "Streptococcus pneumoniae" OR streptococcus pneumonia OR S. pneumonia OR pneumococcal OR "Haemophilus influenzae" OR haemophilus influenza OR NTHi OR Hib OR hemophilus OR haemophilus OR pneumococ) AND (epidemiology OR prevalence OR incidence) [Words] |
| Carriage | (Carrier State OR carriage OR Nose microbiology OR pharynx microbiology OR Nasopharynx microbiology OR Oropharynx microbiology OR colonization OR colonisation OR colonizing OR colonising OR bacterial load OR bacterial density) AND (Respiratory bacterial pathogen OR "Streptococcus pneumoniae" OR streptococcus pneumonia OR S. pneumonia OR pneumococcal OR "Haemophilus influenzae" OR haemophilus influenza OR NTHi OR Hib OR hemophilus OR haemophilus OR pneumococ) AND (epidemiology OR prevalence OR incidence) [Words] |
| Herd Protection | (herd protection OR herd immunity OR indirect protection OR indirect immunity OR herd effect)  AND (Respiratory bacterial pathogen OR "Streptococcus pneumoniae" OR streptococcus pneumonia OR S. pneumonia OR pneumococcal OR "Haemophilus influenzae" OR haemophilus influenza OR NTHi OR Hib OR hemophilus OR haemophilus OR pneumococ) [Words] |
| Other related diseases | (bronchitis OR bronchiolitis OR asthma OR reactive airway disease OR sinusitis OR pharyngitis OR tonsillitis OR conjunctivitis) AND (child OR children OR pediatric OR paediatric) AND (epidemiology OR prevalence OR incidence) [Words] |

**Supplementary Table 6. Search strategy for ScieLO**

| General String | brazil OR brasil OR Brasil OR Brazil OR "brasil" OR "brazil" [All indexes] AND Vaccines OR Pneumococcal Vaccines OR pneumococcal conjugate vaccine OR 10-valent OR PHiD-CV OR PCV10 OR PCV-10 OR Synflorix OR ten-valent OR pneumococcal non-typeable Haemophilus influenzae protein D conjugate vaccine [All indexes] AND 2018 OR 2019 OR 2020 [Publication year] |
| --- | --- |
| Vaccines | Vaccines OR Pneumococcal Vaccines OR pneumococcal conjugate vaccine OR 10-valent OR PHiD-CV OR PCV10 OR PCV-10 OR Synflorix OR ten-valent OR pneumococcal non-typeable Haemophilus influenzae protein D conjugate vaccine [All indexes]  AND 2011 OR 2012 OR 2013 OR 2014 OR 2015 [Publication year] |
| Otitis | (Otitis media OR otitis OR acute otitis media OR AOM OR OM OR OME OR tympanostomy OR myringotomy OR otorrhea OR chronic suppurative otitis media OR CSOM)  AND (epidemiology OR prevalence OR incidence) [All indexes]  AND 2011 OR 2012 OR 2013 OR 2014 OR 2015 [Publication year] |
| Pneumonia | (Pneumonia OR Community-Acquired Infections OR CAP OR lower respiratory tract infection OR respiratory infection)  AND (epidemiology OR prevalence OR incidence) [All indexes]  AND 2011 OR 2012 OR 2013 OR 2014 OR 2015 [Publication year] |
| Invasive Disease | (Invasive Pneumococcal Disease OR IPD OR invasive bacterial disease OR Bacteremia OR bacteremia OR bacteraemia OR Meningitis OR sepsis or empyema OR septicemia OR septicaemia) AND (Respiratory bacterial pathogen OR "Streptococcus pneumoniae" OR streptococcus pneumonia OR S. pneumonia OR pneumococcal OR "Haemophilus influenzae" OR haemophilus influenza OR NTHi OR Hib OR hemophilus OR haemophilus OR pneumococ) AND (epidemiology OR prevalence OR incidence) [All indexes]  AND 2011 OR 2012 OR 2013 OR 2014 OR 2015 [Publication year] |
| Carriage | (Carrier State OR carriage OR Nose microbiology OR pharynx microbiology OR Nasopharynx microbiology OR Oropharynx microbiology OR colonization OR colonisation OR colonizing OR colonising OR bacterial load OR bacterial density) AND (Respiratory bacterial pathogen OR "Streptococcus pneumoniae" OR streptococcus pneumonia OR S. pneumonia OR pneumococcal OR "Haemophilus influenzae" OR haemophilus influenza OR NTHi OR Hib OR hemophilus OR haemophilus OR pneumococ) AND (epidemiology OR prevalence OR incidence) [All indexes]  AND 2011 OR 2012 OR 2013 OR 2014 OR 2015 [Publication year] |
| Herd Protection | (herd protection OR herd immunity OR indirect protection OR indirect immunity OR herd effect)  AND (Respiratory bacterial pathogen OR "Streptococcus pneumoniae" OR streptococcus pneumonia OR S. pneumonia OR pneumococcal OR "Haemophilus influenzae" OR haemophilus influenza OR NTHi OR Hib OR hemophilus OR haemophilus OR pneumococ) [All indexes]  AND 2011 OR 2012 OR 2013 OR 2014 OR 2015 [Publication year] |
| Other related diseases | (bronchitis OR bronchiolitis OR asthma OR reactive airway disease OR sinusitis OR pharyngitis OR tonsillitis OR conjunctivitis) AND (epidemiology OR prevalence OR incidence) AND (child OR children OR pediatric OR paediatric) [All indexes]  AND 2011 OR 2012 OR 2013 OR 2014 OR 2015 [Publication year] |

**Supplementary Table 7. STROBE tool for assessing susceptibility to bias of observational studies [1-3]**

| **Criteria category** | **Domain** | **Tool item must address** | **Risk of Bias^#^  (H, M, L, D)** |
| --- | --- | --- | --- |
| Major* | *Methods for selecting study participants | Appropriate source population (cases, controls and cohorts) and inclusion or exclusion criteria |  |
|  | *Methods for measuring exposure and outcome variables | Appropriate measurement methods for both exposure(s) and/or outcome(s) |  |
|  | *Methods to control confounding | Appropriate design and/or analytical methods |  |
| Minor | Design-specific sources of bias (excluding confounding) | Appropriate methods outlined to deal with any design-specific issues such as recall bias, interviewer bias, biased loss to follow or blinding |  |
|  | Statistical methods (excluding control of confounding) | Appropriate use of statistics for primary analysis of effect |  |

*****Around half of the checklists included what we regard as the three most fundamental domains of appropriate selection of participants, appropriate measurement of variables and appropriate control of cofounding.

**^#^Risk of Bias**

**H** (High risk of bias) clearly indicates bias

**M** (moderate risk of bias) suggest potential bias

**L** (low risk of bias) clearly excludes bias

**?** (Doubtful risk of bias) suggests doubts about potential bias

**Summary judgment of the study: High, Moderate, or Low risk of bias**

- **High risk of bias:** ≥1 of any criteria clearly (H) indicates bias, or ≥2 major criteria suggest potential bias (M) or doubts (?)
- **Moderate risk of bias:** ≥2 of any criteria suggest potential bias (M) or doubts (?) (<2 major criteria)

**Low risk of bias:** Low (L) risk of bias in all major criteria and <2 of minor criteria suggest potential bias (M) or doubts (?)

**Supplementary Table 8. Quality assessment criteria**

| **Study Design** | **Quality of Evidence** | **Lower if** | **Higher if** |
| --- | --- | --- | --- |
| Randomized trial | High | Risk of bias  -1 Serious  -2 Very serious | Large effect  +1 Large  +2 Very large |
|  | Moderate | Inconsistency  -1 Serious  -2 Very serious | Dose response  +1 Evidence of a gradient |
| Observational study | Low | Indirectness  -1 Serious  -2 Very serious | All plausible confounding  +1 Would reduce a demonstrated effect or  +1 Would suggest a spurious effect when results show no effect |
|  | Very Low | Imprecision  -1 Serious  -2 Very serious  Publication bias  -1 Likely  -2 Very likely |  |

**Supplementary Figure 1. GRADE Evaluation – Schematic view of GRADE’s process for developing recommendations [4]**


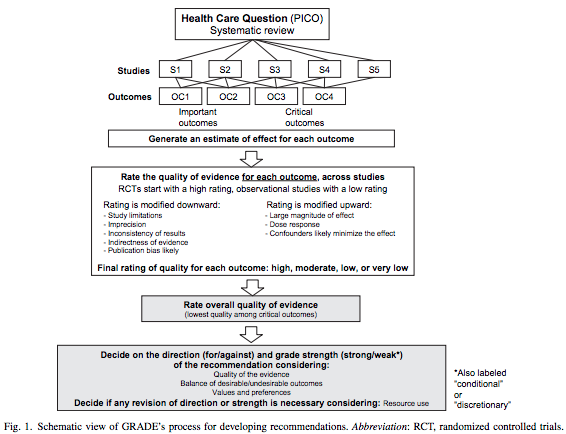


GRADE, Grading of Recommendations Assessment, Development, and Evaluation; OC, outcome; RCT, randomized controlled trial; S, study

Source: Figure has been taken directly from Guyatt et al.[^47^](#_ENREF_47)

**Supplementary Figure 2. Distribution of studies* by outcome and age group**


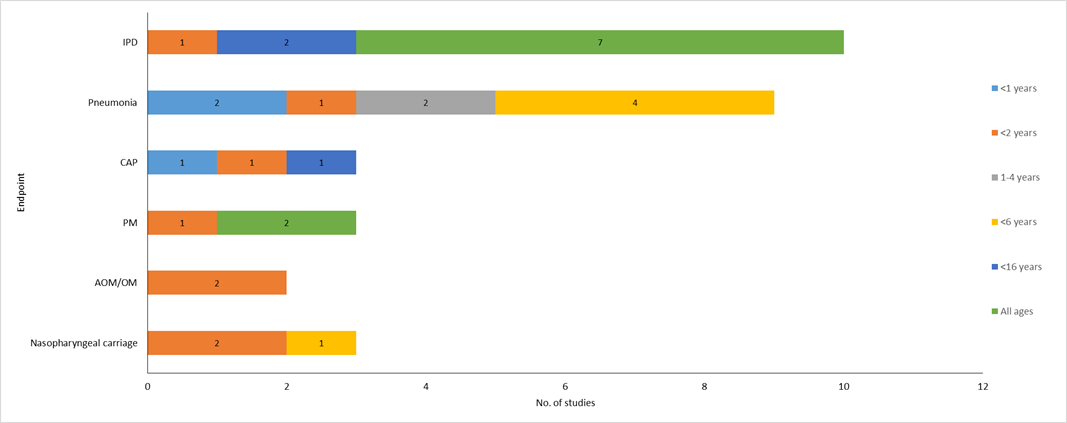


AOM, acute otitis media; CAP, Community-acquired pneumonia; IPD, invasive pneumococcal disease; PM, pneumococcal meningitis; OM, otitis media

*Note: Total N=30 because in 1 publication >1 outcome was reported.

**Supplementary Figure 3. (A) Risk of Bias Assessment using STROBE, (B) Quality of evidence and strength of recommendation using GRADE (N=19*)**

(A)


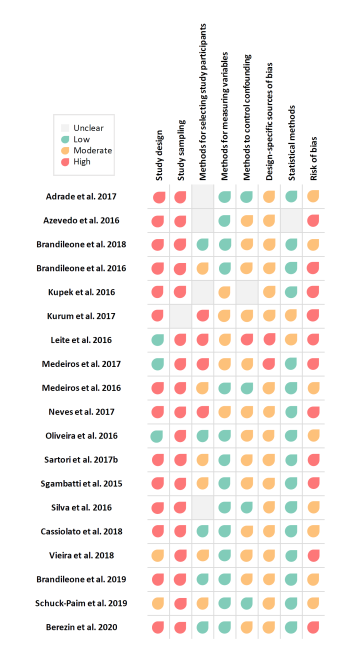


| **Publications (n=19)** | **Study Design** | **Risk of Bias** | **Inconsistency** | **Indirectness** | **Imprecision** | **Publication Bias** | **Quality of Evidence** | **Strength of Recommendation** |
| --- | --- | --- | --- | --- | --- | --- | --- | --- |
| Andrade et al. 2017[^17^](#_ENREF_17) | Time-series analysis (interrupted) | Serious | Serious | Very Serious | Very Serious | Undetected | **Low** | **Strong** |
| Azevedo et al. 2016 [^18^](#_ENREF_18) | Hospital-based surveillance | Serious | Very Serious | Very Serious | Very Serious | Undetected | **Very Low** | **Weak** |
| Brandileone et al. 2018 [^20^](#_ENREF_20) | Laboratory-based surveillance | Serious | Serious | Serious | Serious | Undetected | **High Low** | **Medium** |
| Brandileone et al. 2016 [^21^](#_ENREF_21) | Cross-sectional Survey | Very Serious | Very Serious | Serious | Very Serious | Undetected | **Low** | **Strong** |
| Kupek et al. 2016 [^24^](#_ENREF_24) | Time-series analysis | Serious | Serious | Very Serious | Very Serious | Undetected | **Low** | **Medium** |
| Kurum et al. 2017 [^25^](#_ENREF_25) | Ecological observational study | Very Serious | Very Serious | Serious | Very Serious | Undetected | **Very Low** | **Weak** |
| Leite et al. 2016 [^26^](#_ENREF_26) | Case-series (retrospective and prospective) | Very Serious | Very Serious | Very Serious | Very Serious | Undetected | **Very Low** | **Weak** |
| Medeiros et al. 2017 [^28^](#_ENREF_28) | Retrospective follow-up study | Serious | Serious | Very Serious | Very Serious | Undetected | **Low** | **Weak** |
| Medeiros et al. 2016 [^27^](#_ENREF_27) | Cross-sectional (retrospective) | Very Serious | Serious | Very Serious | Very Serious | Undetected | **Very Low** | **Weak** |
| Neves et al. 2017 [^29^](#_ENREF_29) | Cross-sectional | Very Serious | Very Serious | Very Serious | Very Serious | Undetected | **Very Low** | **Weak** |
| Oliveira et al 2016 [^30^](#_ENREF_30) | Cohort (prospective) | Serious | Serious | Very Serious | Serious | Undetected | **Low** | **Weak** |
| Sartori et al 2017b [^31^](#_ENREF_31) | Time-series analysis (interrupted) | Serious | Serious | Very Serious | Very Serious | Undetected | **Low** | **Strong** |
| Sgambatti et al. 2015 [^33^](#_ENREF_33) | Comparative Study (probabilistic linkage) | Serious | Very Serious | Very Serious | Very Serious | Undetected | **Very Low** | **Weak** |
| Silva et al. 2016 [^34^](#_ENREF_34) | Time-series analysis | Serious | Very Serious | Very Serious | Very Serious | Undetected | **Very Low** | **Medium** |
| Cassiolato et al. 2018 [^23^](#_ENREF_23) | Surveillance | Serious | Serious | Very Serious | Serious | Undetected | **Low** | **Weak** |
| Vieira et al. 2018 [^35^](#_ENREF_35) | Ecological Database Study | Very Serious | Serious | Serious | Serious | Undetected | **Low** | **Medium** |
| Brandileone et al. 2019 [^22^](#_ENREF_22) | Cross-sectional | Serious | Very Serious | Very Serious | Serious | Undetected | **Very Low** | **Weak** |
| Schuck-Paim et al. 2019 [^32^](#_ENREF_32) | Ecological Database Study | Serious | Serious | Serious | Serious | Undetected | **Low** | **Medium** |
| Berezin et al. 2020 [^19^](#_ENREF_19) | Surveillance | Very Serious | Serious | Serious | Serious | Undetected | **Low** | **Medium** |

(B)

*Note: This assessment was performed for full-text publications and not conference abstracts.

**Summary judgment of the study: High, Moderate, or Low risk of bias**

- **High risk of bias:** ≥1 of any criteria clearly (H) indicates bias, or ≥2 major criteria suggest potential bias (M) or doubts (?)
- **Moderate risk of bias:** ≥2 of any criteria suggest potential bias (M) or doubts (?) (<2 major criteria)
- **Low risk of bias:** Low (L) risk of bias in all major criteria and <2 of minor criteria suggest potential bias (M) or doubts (?)

Refer to Supplementary Table 7 for the STROBE tool
